# Ornithine and breast cancer: a matched case–control study

**DOI:** 10.1038/s41598-020-72699-9

**Published:** 2020-09-23

**Authors:** Jiayi Zhang, Baihui Tao, Yiran Chong, Shuang Ma, Gang Wu, Hailong Zhu, Yi Zhao, Shitao Zhao, Mengmeng Niu, Shutian Zhang, Tianyi Wang, Shuman Yang, Wenjing Qiao, Ann M. Vuong, Jincheng Li, Demiao Zhu, Wei Tao

**Affiliations:** 1 Department of Ultrasonography, The First Affiliated Hospital of Jinzhou Medical University, Jinzhou, Liaoning China; 2 Department of Breast Surgery, The First Affiliated Hospital of Jinzhou Medical University, 5-2 Renmin Street, Jinzhou, 121001 Liaoning China; 3 General Surgery/Liver and Pancreas Unit, The First Affiliated Hospital of Jinzhou Medical University, Jinzhou, Liaoning China; 4 Department of Epidemiology and Biostatistics, School of Public Health, Jilin University, Changchun, Jilin China; 5 Department of Epidemiology and Biostatistics, School of Public Health, University of Nevada, Las Vegas, USA

**Keywords:** Cancer, Drug discovery

## Abstract

In vivo and vitro evidence indicates that ornithine and its related metabolic products play a role in tumor development. Whether ornithine is associated with breast cancer in humans is still unclear. We examined the association between circulating ornithine levels and breast cancer in females. This 1:1 age-matched case–control study identified 735 female breast cancer cases and 735 female controls without breast cancer. All cases had a pathological test to ascertain a breast cancer diagnosis. The controls were ascertained using pathologic testing, clinical examinations, and/or other tests. Fasting blood samples were used to measure ornithine levels. The average age for cases and controls were 49.6 years (standard deviation [SD] 8.7 years) and 48.9 years (SD 8.7 years), respectively. Each SD increase in ornithine levels was associated with a 12% reduction of breast cancer risk (adjusted odds ratio [OR] 0.88; 95% confidence interval [CI] 0.79–0.97). The association between ornithine and breast cancer did not differ by pathological stages of diagnosis or tumor grades (all *P* for trend > 0.1). We observed no effect measure modification by molecular subtypes (*P* for interaction = 0.889). In conclusion, higher ornithine levels were associated with lower breast cancer risk in females.

## Introduction

Breast cancer is a major public health problem worldwide as it remains the most common malignancy among females^1^. In the United States (US), the age-standardized incidence rate for breast cancer for 2012–2016 was 126.8 per 100,000 females; the age-standardized death rate due to breast cancer for 2013–2017 was 20.3 per 100,000 females^1^. As compared to US, China has a relatively lower incidence rate for breast cancer, with an age-standardized incidence rate of 29 per 100,000 females^2^. Among Chinese females, the age-standardized mortality rate due to breast cancer was 6.6 per 100,000 in 2011^2^. Although China has a lower breast cancer incidence rate, it is projected that the age standardized incidence rate for breast cancer will increase to 85 per 100,000 females aged 35–69 years in 2021^3^. It is likely that the age-standardized mortality rate of breast cancer will mirror the predicted increase of the breast cancer incidence rate^4^. To prevent and treat breast cancer more effectively, the search for novel breast cancer risk assessments and treatment metabolites is crucial.

Ornithine is a non-essential and nonprotein amino acid; it is involved in many metabolic pathways. Briefly, ornithine is a substrate for enzymes ornithine transcarbomylase (OTC), ornithine aminotransferase (OAT) and ornithine decarboxylase (ODC)^5^. Through OTC, OAT, and ODC, ornithine produces citrulline, proline, and polyamines, respectively^5^. Citrulline plays a key role in the urea cycle. Insufficient levels of citrulline can lead to hyperammonemia^6,7^. Proline is a component in the formation of collagen^8^. Polyamines activate the eukaryotic translation initiation factor 5A (EIF5A), which acts as a translation initiation factor for proteins^9^.

In vivo and vitro evidence suggests that ornithine and its related metabolic products, including ammonia and polyamine, play a role in cancer cell activity as well as tumor development^5,10–12^. However, it is unknown whether ornithine is associated with breast cancer development in humans. Given the heavy burden of breast cancer in the population and its projected increase, determining whether ornithine is related to breast cancer development could potentially assist in the improvement of current breast cancer risk assessments and in the development of innovative therapeutic strategies. Therefore, we examined the association between circulating ornithine levels and breast cancer in females.

## Methods

### Study setting and subjects

All participants in our study were recruited from The First Affiliated Hospital of Jinzhou Medical University, Jinzhou, Liaoning, which is located in the northeastern part of China. The First Affiliated Hospital of Jinzhou Medical University has 82 clinical departments (i.e., Breast Surgery, Liver and Pancreas Surgery) and five medical technology departments (i.e., key laboratory of Liaoning tumor clinical metabolomics). The hospital has more than 2800 beds, and has an annual outpatient and inpatient care of approximately 940,000 and 79,000, respectively. It is considered as one of the major regional medical centers in West Liaoning, providing medical care to approximately 11.2 million people.

This study consisted of inpatients treated by the Department of Breast Surgery during 2015–2018. The inclusion criteria for participants were: (1) being female; (2) having a clear breast cancer or non-breast cancer diagnosis; (3) having a valid ornithine assay available; and (4) having complete and valid data on covariates, including age, body weight, body height, age at menarche, hypertension diagnosis, type II diabetes diagnosis, history of cancer, smoking, alcohol consumption, family history of cancer, postmenopausal status, and parity. Individuals with current and past ornithine treatments were excluded from participation in the study. The study was approved by the institutional review board (IRB; Project #: 202007) at The First Affiliated Hospital of Jinzhou Medical University. Written informed consent was obtained from all participants in the study. All research was performed in accordance with relevant guidelines/regulations.

### Assessment of cases and controls

All female breast cancer cases were ascertained using pathologic testing at the Department of Breast Surgery for the period of 2015–2018. Pathological stages of diagnosis were determined using the 7th edition of the American Joint Committee on Cancer Tumor-Node-Metastasis (TNM) classification method^13^. Histological tumor grades were assessed according to the most recent Chinese guideline for breast cancer^14^. Molecular subtypes of breast cancer, including human epidermal growth factor receptor-2 (HER2)-enriched (estrogen receptors [ER]−, progesterone receptors [PR] −, and HER2+) , luminal A (ER+ and/or PR+ ; HER2−), luminal B (ER+ and/or PR+ ; HER2+), and basal-like (ER−, PR−, and HER2−), were also assessed using established methods^15^. The controls were also selected from inpatients treated by the Department of Breast Surgery during 2015–2018. All controls were confirmed to be free of breast cancer using pathologic testing, clinical examinations and/or other tests (i.e., routine blood test and bacteriological examination). After ascertainment of cases and controls, we individually matched cases to controls by age (± 1 year) at a 1:1 ratio.

### Ascertainment of covariates

The covariates for this study included demographics (age and body mass index [BMI]), lifestyle factors (smoking status and alcohol consumption), and medical history (age at menarche, postmenopausal status, hypertension diagnosis, type II diabetes diagnosis, prior history of cancer, family history of cancer, and parity). Body weight was measured (to the nearest 0.1 kg) with participants standing shoeless and wearing light clothing on an electronic scale. We measured body height to the nearest 0.1 cm using a wall-mounted stadiometer. BMI was calculated using the formula: weight (kg)/(height (m))^2^. A hypertension diagnosis was defined as having a systolic blood pressure ≥ 140 mmHg or a diastolic blood pressure ≥ 90 mmHg (https://www.nhlbi.nih.gov/health/health-topics/topics/hbp/). A type II diabetes diagnosis was assessed based on the established method^16^. Age at menarche, history of cancer, smoking, alcohol consumption, family history of cancer, postmenopausal status, and parity were extracted from medical records. Parity was classified into 0, 1, 2, and 3+.

### Blood collection and processing

Before treating patients, their fasting blood samples (time of fasting > 8 h) were collected from cases and controls with vacutainer tubes in the morning. Using dried blood filter paper, we made dried blood spot samples from the collected fasting blood samples. All dried blood spot samples were stored at − 80 °C until assays were completed.

### Ornithine assay

Measurement of ornithine levels was performed based on previously described methods^17^. Briefly, the dried blood spot papers were punched into 3-mm (diameter) discs. These discs were extracted with ethanol and centrifuged (2 min at 1500*g*) to collect the supernatant; the supernatant was filtered and moved to 96-well plates. Standard ornithine (catalog number: NSK-A; Cambridge Isotope Laboratory, Tewksbury, MA, USA) solution samples served as quality control samples. These plates were dried under 50 °C pure nitrogen flow, incubated with 1-butanol/acetyl chloride mixture and dried again at 50 °C pure nitrogen flow. Lastly, mobile phase solution (80% acetonitrile aqueous solution) was used to dissolve dried samples, which were measured with liquid chromatography coupled with mass spectrometry (high performance liquid chromatography detector LC-20A [Shimadze, Japan] and tandem mass spectrometry detection system AB Sciex 4000 QTrap [AB Sciex, Framinham MA, USA]); this platform is reported to be able to sensitively detect biomolecules^18^.

### Statistical analysis

In the descriptive analysis, the characteristics of participants with and without breast cancer were compared. We also descriptively analyzed the baseline characteristics according to the quartiles of ornithine in the controls.

We used conditional logistic regression models to estimate odds ratios (ORs) and corresponding 95% confidence intervals (CIs) for the associations between 1 standard deviation (SD) increase in ornithine levels and breast cancer. Ornithine levels were transformed into the logarithmic scale to approximate a normal distribution. The final models were adjusted for postmenopausal status and parity. We also considered the following covariates for inclusion in the final model: BMI, age at menarche, hypertension diagnosis, type II diabetes diagnosis, history of cancer, smoking, alcohol consumption, and family history of cancer. However, they failed to meet the criteria of *P* < 0.10 in the univariate analyses with breast cancer and thus were excluded in the final adjustment. We also used conditional logistic regression models to estimate the association between varying levels of ornithine (in quartiles) and breast cancer. Ornithine was categorized into quartiles based on its distribution among the controls. These models also adjusted for postmenopausal status and parity.

In unconditional logistic regression models, subgroup analyses by pathological stages of diagnosis and tumor grades were performed to determine whether the association between ornithine (per 1-SD increase) and breast cancer changed with pathological stages of diagnosis and tumor grades. The trend of the association across pathological stages of diagnosis and tumor grades was tested among cases using multiple linear regression models, in which continuous measures of ornithine was the independent variable and pathological stages of diagnosis and tumor grades were dependent variables. To determine whether molecular subtypes of breast cancer influenced the association, we conducted unconditional multivariable logistic regression to estimate associations between ornithine levels (per 1-SD increase) and breast cancer by molecular subtypes. *P* for interaction between molecular subtypes and ornithine levels were tested using the interaction term (molecular subtypes* ornithine levels [per 1-SD increase]) among cases in unconditional logistic regression models. All unconditional logistic regression and linear regression models were adjusted for age, BMI, age at menarche, hypertension diagnosis, type II diabetes diagnosis, history of cancer, smoking, alcohol consumption, family history of cancer, postmenopausal status, and parity. All statistical analyses were performed utilizing SPSS (version: 24.0; SPSS, Chicago, IL, USA) and R software (Version 3.5.3, R Foundation for Statistical Computing).

## Results

This study included 735 breast cancer cases and 735 non-breast cancer controls. A total of 253 (34.4%), 339 (46.1%), and 143 (19.5%) breast cancer cases had pathologically confirmed diagnosis stages of I, II and III, respectively. There were 50 (6.8%), 447 (60.8%), and 99 (13.5%) cases with tumor grades of I, II and III, respectively; there were 139 cases (18.9%) that did not have information on tumor grades. There were 109 (14.8%), 142 (19.3%), 117 (15.9%), and 341 (46.4%) cases with basal-like, HER2-enriched, Luminal A, and Luminal B molecular subtypes, respectively. Twenty-six (3.5%) cases were not tested for molecular subtypes. A majority of controls (95.0%) had a benign breast lump, while other controls had either a mastitis (3.1%), a benign accessory breast lump (0.7%), hyperplasia of mammary glands (0.5%), a benign axillary lump (0.3%), a benign chest wall lump (0.3%), or a lipoma of the breast (0.1%).

The average age for cases and controls were 49.6 years (SD 8.7 years) and 48.9 years (SD 8.7 years), respectively (Table 1). Cases had a greater proportion of women in a postmenopausal status than controls (40.5% vs. 33.6%). Cases were also more likely to be nulliparous than controls (4.9% vs. 2.5%). Other characteristics, including age, BMI, age at menarche, hypertension diagnosis, type II diabetes diagnosis, history of cancer, smoking status, alcohol consumption and family history of cancer, were not significantly different between cases and controls. There was a negative association between ornithine quartiles and BMI. Age, age at menarche, hypertension diagnosis, type II diabetes diagnosis, history of cancer, smoking status, alcohol consumption, family history of cancer, postmenopausal status, and parity were not associated with ornithine levels (Supplementary Table S1).

**Table 1:** Characteristics for breast cancer cases and non-breast cancer controls.

Variable	Cases (N = 735)	Controls (N = 735)	*P*
Age (years)	49.6 (8.7)	48.9 (8.7)	0.151
Body mass index (kg/m^2^)	24.3 (3.5)	24.3 (3.3)	0.957
Age at menarche (years)	15.2 (1.8)	15.2 (1.7)	0.903
Hypertension diagnosis (n, %)	91 (12.4)	99 (13.5)	0.534
Type II diabetes diagnosis (n, %)	38 (5.2)	26 (3.5)	0.125
History of cancer (n, %)	23 (3.1)	14 (1.9)	0.134
Smoking (n, %)	19 (2.6)	18 (2.5)	0.868
Alcohol consumption (n, %)	2 (0.3)	2 (0.3)	1.000
Family history of cancer (n, %)	44 (6.0)	49 (6.7)	0.592
Postmenopausal status (n, %)	298 (40.5)	247 (33.6)	0.006
**Parity (n, %)**			0.002
0	36 (4.9)	18 (2.5)	
1	483 (65.7)	496 (67.5)	
2	178 (24.2)	203 (27.6)	
3+	38 (5.2)	18 (2.5)	

Unless otherwise specified, variables are presented as the means (standard deviations).

Each SD increase in ornithine levels was associated with decreased odds of breast cancer (OR 0.89; 95% CI 0.80–0.98; Table 2). After adjusting for postmenopausal status and parity, a significant inverse association remained (OR 0.88; 95% CI 0.79–0.97), with a reduction in breast cancer risk of 12% with every SD increase in ornithine levels. When the ornithine levels were classified into quartiles, females with ornithine levels in the highest quartile also had significant lower risk of breast cancer compared to those with ornithine levels in the lowest quartile (adjusted OR 0.70; 95% CI 0.53–0.94).

**Table 2:** Multivariable logistic regression analysis of the association between ornithine levels and breast cancer.

Quartile of ornithine	Range of ORNITHINE (µmol/L)	No. of cases	No. of controls	Unadjusted odds ratio (95% confidence interval)	Adjusted odds ratio (95% confidence interval)^a^
1	< 17.2	201	165	Referent	
2	≥ 17.2; < 24.5	188	181	0.86 (0.65, 1.15)	0.87 (0.65, 1.17)
3	≥ 24.5; < 38.8	173	194	**0.74 (0.56, 0.99)**	**0.73 (0.54, 0.98)**
4	≥ 38.8	173	195	**0.74 (0.56, 0.99)**	**0.70 (0.53, 0.94)**
Overall (Per 1-SD increase)		735	735	**0.89 (0.80, 0.98)**	**0.88 (0.79, 0.97)**

^a^Odds ratios were adjusted for postmenopausal status and parity. Bold values are statistically significant at α = 0.05.

**Figure 1:**
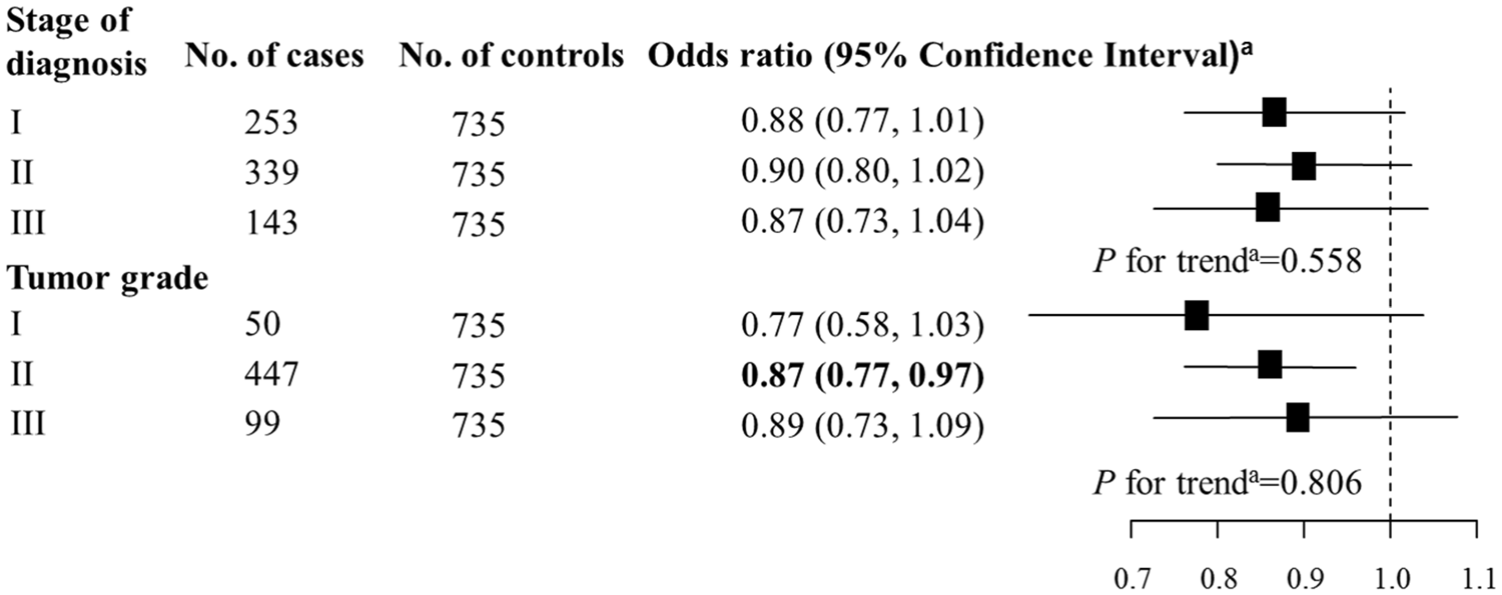
Association between ornithine (per 1-SD increase) and breast cancer by pathological stages of diagnosis and tumor grades. ^a^Odds ratio and *P* for trend were adjusted for age, body mass index, age at menarche, hypertension diagnosis, type II diabetes diagnosis, history of cancer, smoking, alcohol consumption, family history of cancer, postmenopausal status, and parity. Bold values are statistically significant at α = 0.05.

**Figure 2:**
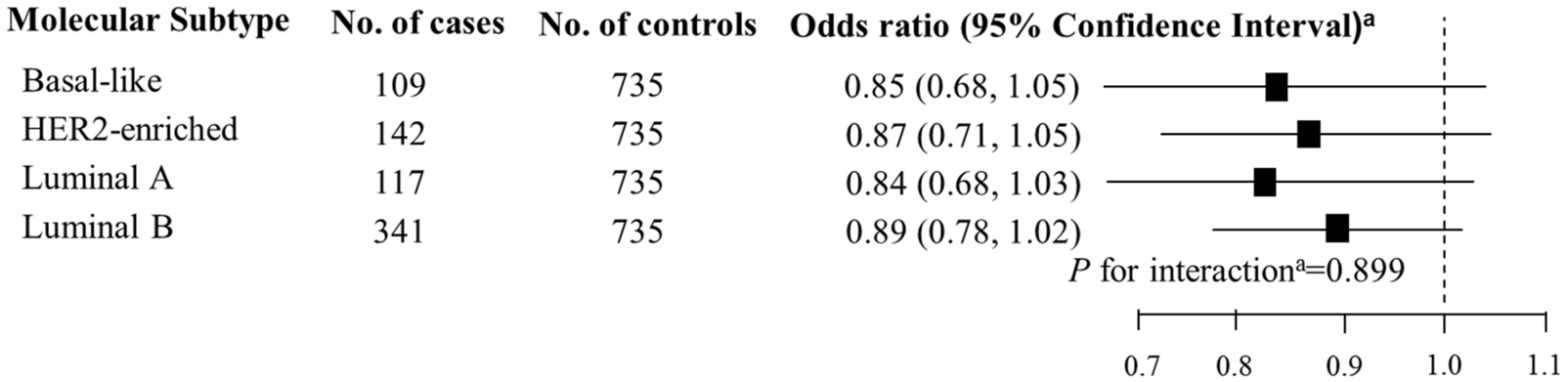
Association between ornithine (per 1-SD increase) and breast cancer by molecular subtypes. ^a^Odds ratio and *P* for interaction were adjusted for age, body mass index, age at menarche, hypertension diagnosis, type II diabetes diagnosis, history of cancer, smoking, alcohol consumption, family history of cancer, postmenopausal status, and parity.

The association between ornithine levels and breast cancer did not differ by pathological stages of diagnosis or tumor grades (all *P* for trend > 0.1; Fig. 1). There was no evidence to support effect measure modification by molecular subtypes of breast cancer (*P* for interaction = 0.889; Fig. 2).

## Discussion

In this age-matched case–control study of Chinese females, we observed a statistically significant negative association between ornithine levels and breast cancer. This association did not differ between pathological stages of diagnosis or tumor grades. Further, there was no evidence to suggest that the relationship differs by molecular subtypes. Given that this is the first human study suggesting a potential protective role of ornithine on breast cancer, future epidemiologic studies are warranted to confirm our research findings.

In our study, we excluded individuals with ornithine treatments. This exclusion is pertinent as it removes the influence of ornithine related drugs on ornithine measurements. In addition, fasting blood samples (time of fasting > 8 h) were collected from cases and controls with vacutainer tubes in the morning. This controlled the impacts of food and the potential circadian rhythm of ornithine levels on ornithine measurements.

The underlying biological mechanisms of action in the relationship between ornithine and breast cancer are still uncertain. On one hand. increased ornithine levels can reduce ammonia and its associated oxidative stress via the urea cycle^19,20^, which may contribute to a protective mechanism as lower oxidative stress levels are associated with lower breast cancer risk^21^. However, ornithine is a substrate for producing polyamine via enzyme ornithine decarboxylase, which promotes the progression of cancer^5,11,22,23^. Although the biological mechanisms of action through which ornithine contributes to carcinogenesis are conflicting, the former mechanism may be dominant based on the findings from the present study.

This research has several clinical and public health implications. Currently, l-ornithine and its mixture of l-asparate are relatively safe and effective drugs for reducing ammonia levels in patients with acute liver failure and hepatic encephalopathy^19,20,24,25^. Our research suggests an additional effect of ornithine for treating breast cancer patients. In addition, if our findings can be replicated by prospective studies, ornithine levels may serve as an independent factor for predicting future breast cancer events. Breast cancer continues to be a major public health problem for females. However, only a few risk factors for breast cancer can be used to prevent and treat the disease effectively. Findings from our study support a potential new target for breast cancer therapy and prediction.

We acknowledge our study has several limitations. First, since this is a case–control study, we cannot make a causal inference about the relationship between ornithine levels and incident breast cancer. Although our research cannot make a causal inference, it provides evidence to support the importance of conducting future studies using a more vigorous study design, including randomized controlled trials [RCTs], for which causal inferences can be made. Second, some risk factors for breast cancer, such as physical activity, hormone replacement (HRT) therapy, and diethylstilbestrol (DES) use were not available. Failure to account for these potential confounders may have influenced our findings. However, it is likely that only a few subjects were on hormone replacement therapy, because only one in five subjects were postmenopausal. Nevertheless, residual confounding may still be an issue. Third, a majority of the controls (95%) for this study had a benign breast lump. Whether using healthy controls will generate similar results remain unclear. Lastly, because this study was performed using hospital-based data, it should be interpreted with caution as our findings may not be generalizable and may be influenced by selection bias.

## Conclusions

High levels of ornithine were observed to be a potential protective factor for breast cancer. Our finding of a significant inverse relationship between ornithine levels and breast cancer in females may have major public health implications regarding breast cancer assessment and treatment methodologies.

## Supplementary information

Supplementary Information.

## Data Availability

The datasets generated and/or analysed during the current study are not publicly available due to ethical reasons but are available from the corresponding author on reasonable request.
