# Supplementary material for: Ornithine and breast cancer: a matched case–control study
Source: Sci Rep. 2020 Sep 23;10:15502. doi: 10.1038/s41598-020-72699-9 (PMC7511971; doi:10.1038/s41598-020-72699-9)
Supplement: Supplementary file 1 — Supplementary Information. [file 41598_2020_72699_MOESM1_ESM.pdf]

Ornithine and Breast Cancer: A Matched Case-Control Study

Jiayi Zhang[a], Baihui Tao[b], Yiran Chong[b], Shuang Ma[b], Gang Wu[b], Hailong Zhu[b],Yi Zhao[b], Shitao Zhao[b], Mengmeng Niu[b], Shutian Zhang[c], Tianyi Wang[c], Shuman Yang[d], Wenjing Qiao[d], Ann M Vuong[e], Jincheng Li[b], Demiao Zhu[b], Wei Tao[b]

[a]Department of Ultrasonography, The First Affiliated Hospital of Jinzhou Medical University, Jinzhou, Liaoning, China; [b]Department of Breast Surgery, The First Affiliated Hospital of Jinzhou Medical University, Jinzhou, Liaoning, China;[c]General Surgery/Liver and Pancreas Unit, The First Affiliated Hospital of Jinzhou Medical University, Jinzhou, Liaoning, China; [d]Department of Epidemiology and Biostatistics, School of Public Health, Jilin University, Changchun, Jilin, China; [e]Department of Epidemiology and Biostatistics, School of Public Health, University of Nevada, Las Vegas, US

Supplementary Table S1. Baseline characteristics according to quartiles of ornithine in controls

	**Quartile 1**	**Quartile 2**	**Quartile 3**	**Quartile 4**	***P* for Trend**[a]
Range of ornithine (μmol/L)	<17.2	≥17.2; <24.5	≥24.5; <38.8	≥38.8	--
N	165	181	194	195	--
Age (years)	48.5 (8.8)	49.5 (9.0)	49.2 (7.7)	48.4 (9.2)	0.169
Body mass index (kg/m^2)	24.4 (3.4)	24.5 (3.2)	24.6 (3.2)	23.7 (3.3)	0.007
Age at menarche (years)	15.2 (1.6)	15.1 (1.6)	15.4 (1.7)	15.1 (1.7)	0.370
Hypertension diagnosis (n, %)	21 (12.7)	27 (14.9)	24 (12.4)	27 (13.9)	0.496
Type II diabetes diagnosis (n, %)	4 (2.4)	12 (6.6)	4 (2.1)	6 (3.1)	0.736
History of cancer (n, %)	4 (2.4)	3 (1.7)	4 (3.1)	3 (4.1)	0.616
Smoking (n, %)	1 (0.6)	3 (0.4)	6 (0.8)	8 (1.1)	0.076
Family history of cancer (n, %)	8 (4.9)	11 (6.1)	19 (9.8)	11 (5.6)	0.876
Postmenopausal status (n, %)	46 (27.9)	61 (33.7)	75 (38.7)	65 (33.3)	0.157
Parity (n, %)					
0	1 (0.6)	6 (3.3)	8 (4.1)	3 (1.5)	0.751
1	118 (71.5)	121 (16.5)	125 (64.4)	132 (67.7)	Referent
2	44 (26.7)	47 (26.0)	57 (29.4)	55 (28.2)	0.491
3+	2 (1.2)	7 (3.9)	4 (2.1)	5 (2.6)	0.584

Unless otherwise specified, variables are presented as the mean (standard deviation). [a]*P* for trend was tested based on quartiles using medians of ornithine as continuous variables, and its model covariates included age, body mass index, age at menarche, hypertension diagnosis, type II diabetes diagnosis, history of cancer, smoking, alcohol consumption, family history of cancer, postmenopausal status, and parity.
